# Unusual catecholaminergic polymorphic ventricular tachycardia and bradycardia caused by a novel triadin variant in 2 siblings from a Malian family

**DOI:** 10.1097/MD.0000000000043596

**Published:** 2025-08-01

**Authors:** Mamadou Diakité, Oumar Samassékou, Koudoussou O. Sanni, Salia Bamba, Bouréma Dembélé, Oumou Traoré, Modibo K. Goita, Alassane Baneye Maiga, Salimata Diarra, Abdoul Karim Sacko, Hamidou O. Ba, Souleymane Coulibaly, Ichaka Menta, Guida Landouré

**Affiliations:** a Faculté de Médecine et d’Odontostomatologie, Université des Sciences, des Techniques et des Technologies de Bamako (USTTB), Bamako, Mali; b Service de Cardiologie, Centre Hospitalier Universitaire Point “G,” Bamako, Mali; c Pediatric Genomics Discovery Program (PGDP), Department of Pediatrics, Yale University School of Medicine, New Haven, CT; d Service de Cardiologie, Centre Hospitalier Universitaire “Gabriel Touré,” Bamako, Mali; e Service de Neurologie, Centre Hospitalier Universitaire Point “G,” Bamako, Mali.

**Keywords:** bradycardia, CPVT, novel variant, *TRDN* gene

## Abstract

**Rationale:**

Catecholaminergic polymorphic ventricular tachycardia (CPVT) is a hereditary arrhythmia syndrome that can cause sudden cardiac death, particularly in young individuals. CPVT is often linked to triadin (*TRDN*) variants that disrupt calcium regulation in the cardiac muscle. Although TRDN-associated CPVT is well documented, its association with resting sinus bradycardia is rarely reported. We describe 2 siblings from a consanguineous Malian family presenting with exertional syncope and bradycardia, in whom a novel homozygous pathogenic TRDN variant was identified.

**Patient concerns:**

The probands, a 17-year-old male and his 11-year-old sister, presented with recurrent exertional syncope beginning at ages 7 and 11, respectively. After obtaining informed consent and assent, a detailed clinical evaluation was performed, including physical examination, electrocardiography, echocardiography, and exercise stress testing. Whole-exome sequencing and segregation analysis were conducted, and variant pathogenicity was assessed using in silico prediction tools.

**Diagnoses:**

Both patients presented sinus bradycardia on electrocardiography and exercise-induced ventricular arrhythmias consistent with a diagnosis of CPVT. Exome sequencing revealed a novel homozygous 5-bp deletion in *TRDN* (NM_006073.4: c.613_617delCAGAA; p.Gln205GlufsTer3), which was classified as pathogenic according to American College of Medical Genetics and Genomics (ACMG) criteria (PVS1, PP5, PM2). The variant co-segregated with the disease in the family.

**Interventions:**

The patients were treated with the nonselective β-blocker nadolol.

**Outcomes:**

Therapy effectively resolved exertional syncope and reduced the frequency of arrhythmic episodes during follow-up.

**Lessons:**

This report highlights an uncommon presentation of bradycardia-associated CPVT caused by a novel *TRDN* variant. To our knowledge, this is the first genetically confirmed case of a *TRDN* variant in sub-Saharan Africa. These findings expand the clinical spectrum of *TRDN*-related cardiac disorders and emphasize the need for genetic exploration of arrhythmic disorders in sub-Saharan Africa, especially for actionable genes.

## 1. Introduction

Catecholaminergic polymorphic ventricular tachycardia (CPVT) is an inheritable arrhythmogenic disorder, which presents as stress-induced bi-directional or polymorphic VT (PVT), and sometimes causes syncope or sudden death in young individuals.^[1,2]^ CPVT is usually due to the presence of pathogenic variants in genes associated with calcium homeostasis, particularly the ryanodine receptor (*RYR2*) and calsequestrin (*CASQ*).^[3]^ Other genes in which variants have been reported include triadin (*TRDN*) but at a substantially lower frequency (<1%).^[4]^ The *TRDN* gene encodes a binding partner of RYR2 and CASQ,^[5]^ playing a crucial role in the calcium release complex within excitable cells. The complex plays an important role in the regulation of intracellular calcium release during excitation–contraction (EC) coupling in cardiomyocytes.^[5]^

Despite the recognition of CPVT and its association with *TRDN* variants in populations around the world,^[6]^ detailed phenotypic and genotypic descriptions of CPVT associated with *TRDN* are rarely reported, especially from Sub-Saharan Africa. Understanding the phenotypic expression of *TRDN* variants in diverse genetic backgrounds is crucial for a comprehensive risk assessment, diagnosis, and management of patients suffering from CPVT. In this report, we describe 2 siblings from a consanguineous Malian family who were diagnosed with CPVT with sinus bradycardia associated with a novel pathogenic homozygous variant in the *TRDN* gene. By sharing these cases, we aim to contribute to the growing body of literature addressing the clinical characteristics and genetic underpinnings of CPVT in diverse populations, emphasizing the necessity for further research in underrepresented regions.

## 2. Methods

This study was done in full compliance with the declaration of Helsinki and approval was obtained from the Ethics Committee of the Faculté de Médecine et d’Odontostomatologie, Université des Sciences, des Techniques et des Technologies de Bamako, Mali. Written informed consent/assent was obtained from all participants with agreement to share and publish the data. The patients benefitted a detailed medical history, physical examination, blood workout, ECG and transthoracic echocardiogram. The diagnosis of CPVT was confirmed by stress test.

**Figure 1.**
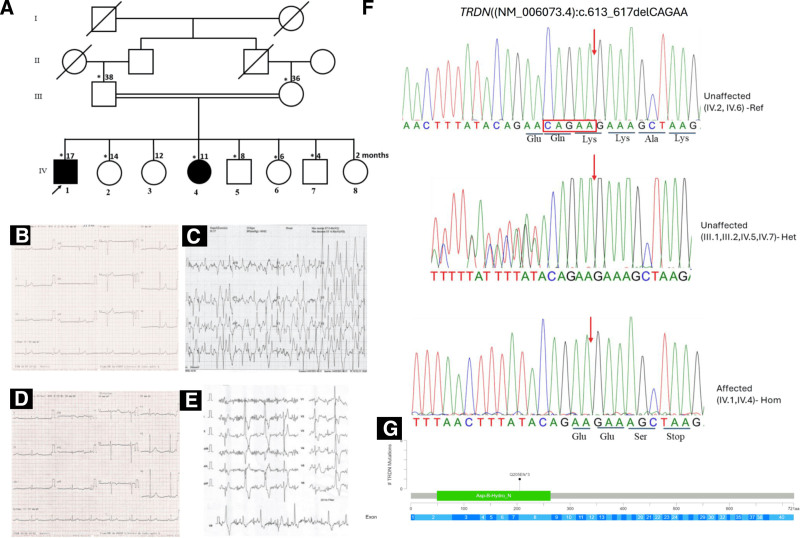
Phenotypic and genotypic findings of the family with CPVT. (A) Pedigree of the family showing a recessive inheritance pattern (arrow indicates the proband and asterisks the participants seen in clinic). (B) Electrocardiographic features of the patient at rest and effort in patient (IV.1) showing bradycardia at rest at 35 bpm. (C) Irregular, non-equipotent bi-directional wide QRS tachycardia at 211 bpm (patient IV.1). (D) Electrocardiographic features of the patient at rest and effort in patient (IV.4) showing bradycardia at rest at 50 bpm. (E) Bi-directional bimorphic extrasystoles (patient IV.4). (F) Chromatogram showing the deletion of 5 nucleotides CAGAA (in red arrow), homozygous in affected siblings (IV.1, IV.4) and heterozygous in both unaffected parents and unaffected sibling brothers (III.1, III.2, IV.5, IV.7) and homozygous wild type (ref) for unaffected sibling sisters (IV.2, IV.3, IV.6). (G) Variant mapping in the protein, Gln205GlufsTer3 is located in functional domain of the triadin. CPVT = Catecholaminergic polymorphic ventricular tachycardia.

DNA was extracted from peripheral blood using the QIAGEN Puregene Blood DNA kit C (Qiagen, Germantown) following the manufacturer’s instruction. Exome sequencing was conducted at the Yale Center for Genomic Analysis (YCGA) through the Pediatric Genomics Discovery Program (PGDP, http://www.yalemedicine.org/departments/pediatric-genomics). Whole exome sequencing (WES) was performed in the participants seen in clinic (III.1, III.2, IV.1, IV.2, IV.4, IV.5, IV.6, IV.7; Fig. 1A). Exomes were captured using IDT xGen exome target capture kit, providing > 96% coverage of RefSeq coding bases with at least 20 independent reads. Enriched libraries were sequenced (2 × 100) on Illumina HiSeq 4000 instruments with an average of 6 gigabases per exome, giving 50× coverage at 90% of reads. Paired-end sequence reads (101 bases) were converted to FASTQ format and were aligned to the reference human genome (hg19). Genetic variants were called by GATK, and they were annotated by ANNOVAR to include information regarding the gene, chromosomal coordinate(s), variants, type of mutation (frameshift, nonsense, nonsynonymous, splicing, and synonymous); and a custom pipeline that includes population allele frequencies, OMIM and ClinVar citations, and numerous *in silico* attributes was used.

Variants were selected based on the suspected inheritance pattern within the family and the number of affected individuals, considering *de novo*, hemizygous, homozygous, and compound heterozygous variants, according to the following criteria: missense, nonsense, frameshift, non-frameshift, or splicing site variants, SNPs with a minor allele frequency of < 0.0005 in the SNP database were selected and filtered. In order to assess deleterious effects of variants, bioinformatics tools were applied including combined annotation dependent depletion (CADD pathogenicity prediction scoring > 20 predicts the top 1% of deleterious variants (https://cadd.gs.washington.edu/home), Sorting Intolerant From Tolerant (SIFT; https://sift.bii.a-star.edu.sg/), MutationTaster (http://www.mutationtaster.org/), protein variation Effect Analyzer (PROVEAN; http://provean.jcvi.org/index.php), and Polymorphism Phenotyping v2 (PolyPhen-2; http://genetics.bwh.harvard.edu/pph2/). The final candidate variant was evaluated according to ACMG (American College of Medical Genetics and Genomics) variant interpretation guidelines and phenotype-driven analysis to identify potential gene-disease associations by leveraging existing knowledge about gene functions and disease phenotypes. Sanger sequencing was performed to confirm the sequence variation and to check for segregation within the family.

## 3. Case description

We report 2 siblings from a consanguineous Malian family of Soninke ethnicity, who present clinical features of catecholaminergic ventricular tachycardia. Their asymptomatic parents and 4 asymptomatic siblings were also enrolled (Fig. 1A).

## 4. Case 1

A 17-year-old male presented with recurrent syncope that began at age 7. The syncopal events were triggered by physical exertion and went unreported until January 2021, and then an ECG revealed sinus bradycardia at 35 bpm following an initial cariological evaluation. Subsequently, following a referral for another cardiological evaluation, the clinical examination was normal except for bradycardia at 35 bpm, with no evident features of myopathy. Initial laboratory investigations, including a complete blood count, renal function tests, and cardiac biomarkers, were within normal limits. A baseline ECG confirmed sinus bradycardia with a heart rate of 35 bpm, a PR interval of 160 ms, a QRS duration of 80 ms, and a corrected QT interval QTc (Fridericia) at 350 ms (Fig. 1B). Transthoracic echocardiography demonstrated normal cardiac structure and function. To assess exertion-induced syncope, an exercise stress test was performed using the Bruce protocol. Initially conducted with a 2-channel Holter monitor, the test revealed isolated monomorphic ventricular extrasystoles at a heart rate of 100 bpm during the second stage of exercise, progressing to more frequent bimorphic ventricular extrasystoles and repetitive salvo-trigeminal extrasystoles at a heart rate of 117 bpm. The ECG at peak exertion showed irregular wide QRS complex tachycardia of varying morphology. A subsequent stress test was performed on a bicycle ergometer with a 12-lead ECG to ensure accurate assessment. The patient developed frequent ventricular extrasystoles again, culminating in sustained bidirectional ventricular tachycardia with a peak rate of 211 bpm (Fig. 1C). The test was terminated to prevent syncope, and the arrhythmia resolved rapidly during recovery.

## 5. Case 2

The second patient is 11-year-old sister, who began experiencing symptoms at the age of 10, characterized by syncopal episodes accompanied by urinary incontinence. The most recent episode occurred 2 months prior to evaluation. These symptoms were not disclosed to medical professionals until the family investigation prompted by her brother’s diagnosis. On clinical examination, she was otherwise asymptomatic except for sinus bradycardia, with a resting heart rate of 50 bpm. Her ECG confirmed the bradycardia but showed no other abnormalities (Fig. 1D). Her exercise stress test was performed using the same protocol as her brother’s. The test revealed a single monomorphic premature ventricular contraction (PVC) with a right inferior axis delay at a heart rate of 112 bpm during the fourth stage of exercise. This was followed by bimorphic, bi-directional PVCs (Fig. 1E), which persisted throughout the test but did not progress to sustained ventricular tachycardia. The test was stopped at 62% of her predicted maximum heart rate due to exhaustion. We diagnosed CPVT based on her exertional syncope and the findings from the exercise test, despite not reaching the threshold for sustained tachycardia.

Both patients were started on oral nadolol, with the dose adjusted according to body surface area (160 mg per day). Following the initiation of treatment, they remained asymptomatic, with stable resting bradycardia and no exertion-induced syncope.

To investigate the genetic basis of CPVT in these patients, we conducted whole exome sequencing (WES) on both affected individuals, as well as their parents and 4 siblings (2 brothers and 2 sisters). Exome sequencing revealed a pathogenic frameshift variant in the *TRDN* gene caused by deletion of 5 nucleotides (c.613_617delCAGAA, NM_006073.4), leading to a premature stop codon (p.Gln205GlufsTer3; NP_006064.2). Both affected siblings were homozygous for this mutation, while the unaffected parents and 2 unaffected sibling brothers were confirmed to be heterozygous carriers of the deletion and 2 unaffected sisters were homozygous for the reference allele.

We confirmed the exome sequencing data by Sanger sequencing (Fig. 1F). This variant has been classified as pathogenic according to the ACMG criteria (PVS1, PP5, PM2).

## 6. Discussion

In this study, we report a novel pathogenic variant of the *TRDN* gene, and catecholaminergic polymorphic ventricular tachycardia (CPVT) associated with sinus bradycardia in 2 siblings from a consanguineous Malian family. This variant is a 5-bp deletion (c.613_617delCAGAA) leading to frameshift and a premature stop codon p.(Gln205GlufsTer3). This finding is not only significant due to the originality of the variant, but also because it represents one of the first genetic associations with CPVT identified in Sub-Saharan Africa.

The occurrence of sinus bradycardia in both patients, particularly at rest, is noteworthy. Although bradycardia has been previously reported in patients with *RYR2* variants,^[7]^ its presence in individuals with CPVT due to *TRDN* variants, as shown in this report, is not well documented. The mechanism underlying this bradycardia is unclear but could reflect early conduction system involvement, secondary ion channel dysfunction, or altered autonomic regulation due to specific genetic variants. Given the resting bradycardia and exertional ventricular tachycardia observed in both siblings, we hypothesize that the observed bradycardia may be potentially associated with pathogenic variants in other genes involved in CPVT or be an extension of *TRDN*-associated CPVT spectrum.

A key finding of this study is the identification of a novel 5-bp deletion (c.613_617delCAGAA) in exon 8 of the *TRDN* gene, which results in a truncated polypeptide at position 208 (Gln205Glufs*3). This variant was found to be homozygous in both affected individuals and heterozygous in 2 siblings and the parents. The location of the deletion within a critical functional domain, specifically the AspB Hydro N region of the TRDN protein, suggests that this variant may compromise the protein’s structural integrity. Previous studies have reported 17 unique *TRDN* variants associated with CPVT across 27 patients from 21 families.^[8]^ Of these, 2 variants, also located in exon 8, were described, underscoring the potential role of this exon as a mutational hotspot. Specifically, 1 variant (c.613C > T, Gln205*) was found in a compound heterozygous state in 4 individuals from 2 French families,^[9]^ while another (c.618delG) was observed in a homozygous state in a Hispanic family, resulting in a truncated polypeptide (p.Ala208fs*15).^[10]^ The novel variant we identified in this region reinforces the importance of exon 8 as a hotspot for *TRDN* mutations in CPVT.

Our findings suggest that this novel variant may have a high frequency in Mali, a hypothesis reinforced by the region’s high prevalence of consanguinity. The observation that 2 out of 6 siblings in our cohort were heterozygous carriers (with 2 siblings untested) further supports this possibility. This underscores the importance of investigating the prevalence of this variant, not only in Mali but also across broader African populations. Establishing genetic counseling and screening programs could be instrumental in facilitating early diagnosis and timely intervention for individuals at risk of CPVT and other arrhythmias. Furthermore, the implications of this finding are significant for the understanding of *TRDN*-related disorders, as it suggests that the phenotypic spectrum may extend beyond established features. The uniqueness of this case emphasizes the importance of genetic evaluation in families with arrhythmia syndromes, particularly in resource-limited settings such as Africa where genetic studies are limited.

## 7. Conclusion

This is the first study that describes a novel *TRDN* variant associated with CPVT in Mali and Sub-Saharan Africa. Our findings extend the clinical and genetic understanding of *TRDN*-related CPVT and highlight the urgent need for further research to enhance the management and prevention of this life-threatening condition.

## Author contributions

**Conceptualization:** Mamadou Diakité, Oumar Samassékou, Guida Landouré.

**Data curation:** Mamadou Diakité, Koudoussou O. Sanni, Salia Bamba, Bouréma Dembélé, Oumou Traoré, Modibo K. Goita, Salimata Diarra, Hamidou O. Ba, Souleymane Coulibaly, Ichaka Menta, Guida Landouré.

**Formal analysis:** Salia Bamba, Oumou Traoré, Salimata Diarra.

**Funding acquisition:** Guida Landouré.

**Investigation:** Mamadou Diakité, Oumar Samassékou, Koudoussou O. Sanni, Bouréma Dembélé, Salimata Diarra, Abdoul Karim Sacko, Hamidou O. Ba, Souleymane Coulibaly.

**Methodology:** Mamadou Diakité, Salia Bamba, Modibo K. Goita, Hamidou O. Ba, Guida Landouré.

**Project administration:** Alassane Baneye Maiga, Guida Landouré.

**Resources:** Oumar Samassékou, Guida Landouré.

**Supervision:** Mamadou Diakité, Oumar Samassékou, Hamidou O. Ba, Souleymane Coulibaly, Ichaka Menta, Guida Landouré.

**Validation:** Mamadou Diakité, Salia Bamba

**Visualization:** Oumar Samassékou.

**Writing – original draft:** Mamadou Diakité, Oumar Samassékou, Koudoussou O. Sanni, Guida Landouré.

**Writing – review & editing:** Koudoussou O. Sanni, Salia Bamba, Bouréma Dembélé, Modibo K. Goita, Abdoul Karim Sacko, Hamidou O. Ba, Souleymane Coulibaly, Ichaka Menta, Guida Landouré.
